# The treatment of migraine patients within chiropractic: analysis of a nationally representative survey of 1869 chiropractors

**DOI:** 10.1186/s12906-017-2026-3

**Published:** 2017-12-04

**Authors:** Craig Moore, Jon Adams, Andrew Leaver, Romy Lauche, David Sibbritt

**Affiliations:** 1 Australian Research Centre in Complementary and Integrative Medicine (ARCCIM), University of Technology Sydney, Level 8, Building 10, 235-253 Jones Street, Ultimo, NSW 2007 Australia; 2 Faculty of Health Science, University of Sydney, Sydney, Australia

**Keywords:** Chiropractic, Migraine, Headaches, Practice-based research network, Utilisation, Manual therapy, Prevalence

## Abstract

**Background:**

While the clinical role of manual therapies in migraine management is unclear, the use of chiropractors for this condition is considerable. The aim of this study is to evaluate the prevalence and characteristics of chiropractors who frequently manage patients with migraine.

**Methods:**

A national cross-sectional survey of chiropractors collected information on practitioner characteristics, clinical management characteristics and practice settings. A secondary analysis was conducted on 1869 respondents who reported on their migraine caseload to determine the predictors associated with the frequent management of patients with migraine.

**Results:**

A large proportion of chiropractors report having a high migraine caseload (HMC) (*n* = 990; 53.0%). The strongest factors predicting a chiropractor having a HMC include the frequent treatment of patients with axial neck pain (OR = 2.89; 95%CI: 1.18, 7.07), thoracic pain (referred/radicular) (OR = 2.52; 95%CI: 1.58, 3.21) and non-musculoskeletal disorders (OR = 3.06; 95%CI: 2.13, 4.39).

**Conclusions:**

Several practice-setting and clinical management characteristics are associated with chiropractors managing a HMC. These findings raise key questions about the therapeutic approach to chiropractic migraine management that deserves further examination. There is a need for more primary research to assess the approach to headache and migraine management provided by chiropractors and to understand the prevalence, burden and comorbidities associated with migraine found within chiropractic patient populations. This information is vital in helping to inform safe, effective and coordinated care for migraine sufferers within the wider health system.

**Electronic supplementary material:**

The online version of this article (10.1186/s12906-017-2026-3) contains supplementary material, which is available to authorized users.

## Background

Migraine is the seventh leading cause of years lived with disability (YLDs) and a common neurological disorder [1]. During an attack, migraine symptoms are characterised by severe, throbbing, unilateral headaches associated with nausea, vomiting, photophobia and/or phonophobia and aggravation from physical activity and while less common, a migraine with aura is further associated with visual, sensory or speech related symptoms [2]. A variety of precipitating factors have been associated with triggering a migraine attack. Triggers reported include weather, stress, poor or over-sleeping, odours, missing meals and certain foods, menses and neck pain [3, 4].

Uncertainty remains regarding the mechanisms associated with the initiation of migraine pain. Evidence suggests migraine pain has a central origin involving the cortex and brainstem [5, 6]. Indirect evidence also suggests migraine pain has a peripheral origin whereby peripheral input from within cervical spine structures causes sensitization of trigeminal nociceptive pathways [7–9]. This may be more common in sufferers with neck pain and may involve convergent nociceptive input via the trigeminal nerve and the upper cervical afferents to the trigeminal cervical complex [10–12]. Interpretation of this indirect evidence may have implications for the role of manual therapies in the treatment of migraine [13, 14]. To date however, clinical trials to support the effectiveness of manual therapies, including soft tissue therapies, spinal manipulation and spinal mobilisation, for the prevention of migraine remains limited, of poor quality and sometimes conflicting [15–17]. Despite this clinical uncertainty, physical therapies, which may include manual therapies, are reported as the most frequently used complementary and alternative therapies for the management of headaches worldwide [18].

Chiropractors are one of the most common complementary and alternative medicine (CAM) providers globally [19–21]. The use of chiropractic for the treatment of headaches appears to be substantial [22–24] with migraine likely to be one of the most common headache types chiropractors manage [25–27]. Consequently, there is a need to better understand how many chiropractors have a high migraine caseload and whether this is more common to a particular type of chiropractor. While the treatment of migraine by chiropractors may be substantial, no research to date has reported on how prevalent such treatment is within the profession or the features of those chiropractors who provide it. In response, this study aimed to investigate the proportion of Australian chiropractors with a high migraine caseload; and the practitioner characteristics, practice characteristics and clinical management factors associated with frequent management of patients with migraine by chiropractors.

## Methods

The analyses presented in this paper were drawn from a questionnaire distributed during recruitment for a national practice-based research network (PBRN) titled the Australian Chiropractic Research Network (ACORN) project. This national project is independently designed and conducted by senior researchers at the Australian Research Centre in Complementary and Integrative Medicine (ARCCIM), University of Technology Sydney. The ACORN 21-item questionnaire examining practitioner, practice and clinical management characteristics was distributed to all registered chiropractors across Australia (approval # 2014000027) [28]. The secondary analyses sub-study reported in this paper were undertaken following ethical approval from the Human Research Ethics Committee of the University of Technology Sydney (approval # ETH16–0474).

### Recruitment and sample

Recruitment for the ACORN PBRN occurred through a profession-wide recruitment strategy conducted from March through to June 2015. An invitation pack was distributed to all registered Australian chiropractors who were invited to both complete the baseline ACORN questionnaire and to consent to participate in the ACORN PBRN project. Distribution was via post (hard copy), email (survey link) and at several regional profession-based conferences and was also made available through the official ACORN website (SurveyGizmo™). The invitation pack was similarly re-distributed with four reminders starting 4 weeks after the initial invitation [28].

A total of 2005 chiropractors (43% of the 4684 Australian chiropractors registered at time of recruitment) completed the baseline ACORN practitioner questionnaire. Participants were generally representative of the wider profession with regards to a number of key indicators when compared to registered chiropractors identified by AHPRA (Australian Health Practitioner Regulation Agency) at the time of recruitment [29] including age (*p* = 0.065) and gender (*p* = 0.634). While the ACORN baseline sample is also generally representative of the wider chiropractic population regarding practice location, we found slight differences in terms of the distribution by location with the questionnaire sample slightly over-represented by chiropractors from South Australia, the Australian Capital Territory, Tasmania and the Northern Territory (*p* < 0.01) [28].

### Instrument

The ACORN questionnaire collected information across three key domains (see Additional file 1). The first was practitioner characteristics (age, gender, education, professional qualifications and memberships in professional associations, years in private practice and professional roles in education, research and other professional areas). The second domain was practice characteristics (average patient care hours, number of weekly patient visits, place, number and type of practice location(s), types of health professionals working in the chiropractor’s practice location, professional referral relationships and use of diagnostic imaging and electronic records). The third domain was clinical management characteristics where all response categories were on a four-point Likert frequency scale (‘never’, ‘rarely’, ‘sometimes’ or ‘often’). This domain was divided into five sub-sections including frequency with which chiropractors discuss listed aspects of health promotion in their care plans; treat patients presenting with a range of listed conditions; treat patient subgroups and utilise listed treatment methods and interventions.

### Statistical analyses

Statistical analyses were conducted using statistical software Stata 13.1 and SPSS 22.0 on those chiropractors who provided an answer to the question on how often they treat patients with migraine (*n* = 1869; 93.2% of all questionnaire respondents). The dependent variable was the frequency of treatment of patients with migraine; ‘never’, ‘rarely’, ‘sometimes’ or ‘often’, which was dichotomized into those who treat patients with migraine ‘often’ and those who treat patients with migraine ‘less often’ (represented by the ‘never’, ‘rarely’ and ‘sometimes’ responses). Data are presented as means and standard deviations, or absolute and relative frequencies.

The bivariate associations between all survey items and the outcome variables were firstly explored using Student’s t-test or chi-square tests, where applicable. Independent predictors of frequency of treating patients with migraine were identified using multiple logistic regression analysis. ACORN survey items with associations from the bivariate analyses (*p* ≤ 0.25) were included in the regression model. A backward stepwise procedure employing a likelihood ratio test was chosen to determine the independent predictors of chiropractors who treat patients with migraine ‘often’. Statistical significance was set at *p* < 0.05. Odds ratios were reported with 95% confidence intervals.

## Results

Of the 1869 chiropractors, 62% were male with a mean (SD) age of 42.1 (12.1) years and most had a Bachelor or Master’s degree qualifications (96%). Participants had worked for an average of 15.8 (11.3) years in practice and worked an average of 27.3 (12.6) patient care hours each week. The majority of chiropractors reported managing patients with migraine ‘often’ (*n* = 990; 53.0%). Fewer participants reported managing patients with migraine ‘sometimes’ (*n* = 765; 40.9%) and only a small percentage reported managing patients with migraine ‘rarely’ (*n* = 106; 5.7%) or ‘never’ (*n* = 8; 0.4%).

Chiropractors with a high migraine caseload (‘often’ group) were more often older (*p* = 0.001), had more years in practice (*p* < 0.001), worked a greater number of patient-care hours per week (*p* < 0.001) and reported a greater number of patient visits per week (*p* < 0.001) than those chiropractors with a lower migraine caseload (Table 1). The practice setting of chiropractors with a high migraine caseload was more often rural (*p* = 0.017) and they less often shared their practice location with a GP (*p* = 0.046) or psychologist/counsellor (*p* = 0.043) while more often had a referral relationship with an occupational therapist (*p* = 0.016), podiatrist (p = 0.016) and/or exercise physiologist (*p* = 0.031). Additionally, these chiropractors more often used imaging in their practice (*p* < 0.001) but less often had diagnostic ultrasound on site (*p* = 0.008) than those chiropractors with a lower migraine caseload (Table 2).

**Table 1:** Distribution of practitioner characteristics across frequency of practitioner treating patients with migraine

Characteristic	Treat patients with migraine		
	Never/rarely/ sometimes (*n* = 879)	Often (*n* = 990)	*p*-value
Age in years (mean ± sd)	41.3 ± 11.7	43.1 ± 12.3	0.001
Gender			
male n (%)	531 (60.7)	624 (63.4)	0.237
female n (%)	344 (39.3)	361 (36.6)	
Qualification n (%)			
Diploma n (%)	20 (2.3)	21 (2.1)	0.718
Advanced diploma n (%)	6 (0.7)	8 (0.8)	
Bachelor n (%)	304 (34.9)	344 (35.0)	
Doctor of Chiropractic n (%)	245 (28.1)	296 (30.1)	
Masters n (%)	288 (33.0)	308 (31.4)	
PhD n (%)	9 (1.0)	5 (0.5)	
Years in practice (mean ± sd)	14.9 ± 11.0	16.8 ± 11.6	< 0.001
Patient care hours/week (mean ± sd)	26.0 ± 11.2	28.0 ± 10.4	< 0.001
Patient visits/week (mean ± sd)	78.1 ± 53.8	95.5 ± 59.2	< 0.001

**Table 2:** Distribution of practice characteristics across frequency of practitioner treating patients with migraine

Characteristic	Treat patients with migraine		*p*-value
	Never/rarely/ sometimes (*n* = 879)	Often (*n* = 990)	
Location			
Urban n (%)	685 (79.6)	727 (74.9)	0.017
One location only	214 (24.5)	257 (26.0)	0.441
Other health professionals in practice location			
General practitioner	68 (7.7)	54 (5.5)	0.046
Podiatrist	93 (10.6)	86 (8.7)	0.165
Medical specialist	26 (3.0)	25 (2.5)	0.567
Physiotherapist	85 (9.7)	91 (9.2)	0.724
Chiropractor	504 (57.3)	595 (60.1)	0.226
Exercise physiologist	56 (6.4)	69 (7.0)	0.605
Psychologist	126 (14.3)	111 (11.2)	0.043
Occupational therapist	17 (1.9)	31 (3.1)	0.102
Referral relationships			
General practitioner	483 (54.9)	581 (58.7)	0.103
Psychologist	119 (13.5)	147 (14.8)	0.418
Physiotherapist	259 (29.5)	329 (33.2)	0.080
Occupational therapist	59 (6.7)	97 (9.8)	0.016
Podiatrist	323 (36.7)	418 (42.2)	0.016
Medical specialist	129 (14.7)	168 (17.0)	0.176
Exercise physiologist	120 (13.7)	171 (17.3)	0.031
Using imaging at least often	332 (38.1)	549 (55.7)	< 0.001
Having imaging on site			
X-ray	138 (15.7)	144 (14.5)	0.487
Magnetic resonance imaging (MRI)	36 (4.1)	26 (2.6)	0.077
Surface electromyography (SEMG)	30 (3.4)	50 (5.1)	0.081
Diagnostic ultrasound	35 (4.0)	19 (1.9)	0.008
Thermography	33 (3.8)	55 (5.6)	0.067

Table 3 displays the clinical management characteristics of chiropractors with a high migraine caseload. The clinical management plans of chiropractors with a high migraine caseload more often included advice on diet/nutrition (*p* < 0.001), smoking/drugs/alcohol (*p* < 0.001), physical activity (*p* = 0.005), occupational health and safety (*p* < 0.001), pain counselling (*p* < 0.001), nutritional supplements (*p* < 0.001) and medications (including for pain/inflammation) (*p* < 0.001) than those chiropractors who less often managed patients with migraine. In addition, those chiropractors with a high migraine caseload more often treated patients presenting with neck, thoracic and low back pain, upper and lower limb disorders, postural disorders, degenerative conditions (all *p* < 0.001), non-musculoskeletal conditions (*p* < 0.001), other headache disorders (excluding migraine) including cervicogenic and tension type headaches (*p* < 0.001) and spine health maintenance/prevention (*p* < 0.001) than chiropractors with a lower migraine caseload. In addition, they were more likely to treat pregnant women (*p* < 0.001), athletes/sports people (*p* < 0.001), Aboriginal and Torres Strait Islander people (ATSI) (*p* < 0.012), patients with work injuries (*p* < 0.001) and traffic injuries (*p* < 0.001), patients from non-English speaking ethnic groups (*p* < 0.035), people receiving post-surgical rehabilitation (*p* < 0.001), and younger and older patients (all *p* < 0.001) than those chiropractors with a lower migraine caseload. The treatment techniques/methods more often used by chiropractors with a high migraine caseload were high velocity, low amplitude (HVLA) spinal manipulation (*p* = 0.023), drop-piece techniques (*p* = 0.015), sacro-occipital techniques (*p* < 0.001), instrument adjusting (*p* = 0.001), biophysics (*p* = 0.040), applied kinesiology (*p* = 0.001), functional neurology (*p* < 0.001), dry needling (*p* = 0.006), heat/cryotherapy (*p* = 0.002), orthotics (*p* < 0.001) and extremity joint manipulation methods (*p* < 0.001).

**Table 3:** Distribution of clinical management characteristics across frequency of practitioner treating patients with migraine

Characteristic	Treat patients with migraine		*p*-value
	Never/rarely/ sometimes (*n* = 879)	Often (*n* = 990)	
Care plan includes (discussed often)			
Diet/nutrition	379 (43.2)	565 (57.4)	< 0.001
Smoking/drugs/alcohol	171 (19.5)	295 (30.1)	< 0.001
Physical activity/fitness	724 (82.8)	861 (87.5)	0.005
Occupational health and safety	325 (37.4)	439 (44.8)	0.001
Pain counselling	175 (20.2)	285 (29.3)	< 0.001
Nutritional supplements	261 (29.8)	435 (44.1)	< 0.001
Medications (including pain/inflammation)	165 (19.1)	264 (27.0)	< 0.001
Conditions (treated often)			
Neck pain: Axial	780 (88.8)	967 (97.8)	< 0.001
Neck pain: Referred/radicular	374 (42.5)	799 (80.7)	< 0.001
Thoracic pain: Axial	654 (74.8)	922 (93.4)	< 0.001
Thoracic pain: Referred/radicular	227 (26.1)	632 (64.4)	< 0.001
Low back pain: Axial	793 (90.5)	968 (98.2)	< 0.001
Low back pain: Referred/radicular	600 (68.5)	910 (92.2)	< 0.001
Lower limb musculoskeletal disorders	395 (45.0)	729 (73.8)	< 0.001
Upper limb musculoskeletal disorders	416 (47.4)	748 (76.1)	< 0.001
Postural disorders	442 (50.5)	765 (77.7)	< 0.001
Degenerative spine conditions	642 (73.1)	986 (99.7)	< 0.001
Headaches (tension, cervicogenic)	642 (73.0)	986 (100.0)	< 0.001
Migraine disorders			
Spine health maintenance/prevention	529 (60.3)	834 (84.8)	< 0.001
Non-Musculoskeletal conditions	106 (16.8)	306 (41.2)	< 0.001
Patient groups (treated often)			
Child: <4 years	198 (22.7)	362 (36.8)	< 0.001
4–18 years	363 (41.6)	627 (63.6)	< 0.001
Older: >65 years	574 (65.8)	794 (80.6)	< 0.001
Aboriginal and Torres Strait islander	8 (0.9)	24 (2.5)	0.012
Pregnant women	233 (26.8)	448 (45.7)	< 0.001
Athletes/sports people	339 (39.1)	572 (58.5)	< 0.001
Work Injuries	250 (38.9)	418 (42.8)	< 0.001
Traffic Injuries	58 (6.7)	196 (20.1)	< 0.001
Post-Surgical Rehabilitation	32 (3.7)	88 (9.0)	< 0.001
Non-English Speaking ethnic groups	43 (5.1)	72 (7.5)	0.035
Techniques/methods (used often)			
Drop-piece	443 (51.0)	549 (56.7)	0.015
Pelvic blocking/sacro-occipital	343 (39.7)	465 (48.1)	< 0.001
Instrument Adjusting	420 (48.4)	545 (56.0)	0.001
Chiropractic Biophysics	28 (3.3)	49 (5.4)	0.040
HVLA manipulation/mobilisation	694 (80.0)	821 (84.1)	0.023
Applied kinesiology	113 (13.1)	182 (19.1)	0.001
Flexion-Distraction	65 (7.6)	81 (8.5)	0.472
Functional Neurology	71 (8.4)	168 (17.8)	< 0.001
Extremity Manipulation	443 (50.9)	648 (66.5)	< 0.001
Musculoskeletal Interventions (used often)			
Dry Needle or acupuncture	98 (11.3)	153 (15.7)	0.006
Soft tissue therapies	573 65.9	650 (66.1)	0.905
Electro-modalities	71 (8.6)	103 (10.6)	0.147
Heat/cryotherapy	118 (13.7)	184 (18.9)	0.002
Orthotics	55 (6.4)	134 (13.8)	< 0.001
Exercise therapy/rehabilitation	411 (47.7)	497 (51.1)	0.140

Logistic regression analysis identified a range of factors independently associated with the likelihood of a chiropractor having a high migraine caseload. These factors included the chiropractor often discussing medications with their patients (including for pain/inflammation) (OR = 1.55; 95%CI: 1.09, 2.21), treating patients with neck pain (axial) (OR = 2.89; 95%CI: 1.18, 7.07), neck pain (referred/radicular) (OR = 1.88; 95%CI: 1.28, 2.77), thoracic pain (referred/radicular) (OR = 2.52; 95%CI: 1.58, 3.21), low back pain (referred/radicular) (OR = 1.78; 95%CI: 1.11, 2.85), upper limb musculoskeletal disorders (shoulder, elbow, wrist, hand) (OR = 1.67; 95%CI: 1.20, 2.31), providing spinal health maintenance/prevention (OR = 1.59; 95%CI: 1.12, 2.25), treating non-musculoskeletal disorders (OR = 3.06; 95%CI: 2.13, 4.39), treating athletes/sports people (OR = 1.65; 95%CI: 1.22, 2.23), employing functional neurology methods in their patient management (OR = 1.63; 95%CI: 1.02, 2.61) and less often having a psychologist/counsellor located in the same practice as the chiropractor (OR = 0.53; 95%CI: 0.34, 0.86) (Table 4).

**Table 4:** Logistic regression output for chiropractors that treat migraine often compared to never/rarely/sometimes

Factors	Odds Ratio	95% C.I.	*p*-value
Non-musculoskeletal disorders	3.058	2.132, 4.388	< 0.001
Neck pain (Axial)	2.889	1.181, 7.068	0.020
Thoracic pain (Referred/radicular)	2.252	1.580, 3.210	< 0.001
Neck pain (Referred/radicular)	1.881	1.280, 2.764	0.001
Low back pain (Referred/radicular)	1.783	1.115, 2.851	0.016
Upper limb Musculoskeletal disorders	1.668	1.206, 2.308	0.002
Athletes or Sports people	1.653	1.225, 2.231	0.001
Functional Neurology	1.632	1.020, 2.610	0.041
Spinal health maintenance/prevention	1.586	1.116, 2.252	0.010
Discussing medication (Including pain/inflammation)	1.555	1.093, 2.213	0.014
Psychologist/counsellor in same practice	0.543	0.342, 0.862	0.010

## Discussion

### Prevalence of migraine management

Our study found a large proportion of Australian chiropractors report managing a high migraine caseload. This appears to support previous studies which have identified a high prevalence of headache in chiropractic patient populations (4.6% - 15.4%) [30–32] and a high prevalence of chiropractic use within the general migraine population (10%–29%) [23, 25, 26, 33]. The high use of chiropractors by those with migraine would suggest these providers are likely to be addressing some of the healthcare needs of this population and raises several questions for further research enquiry.

For instance, there is a need to better understand all of the relevant patient management approaches included within chiropractic migraine management and whether these approaches vary from those reported in routine Australian chiropractic practice which favours spinal manipulation, soft tissue therapy and exercise prescription [34]. For instance, while management of public health and lifestyle factors, have been captured in recent chiropractic workforce data [35, 36] there has been no detailed examination on how these aspects of patient management are utilised in the management of migraine. For example, little is known about the role chiropractors play in patient education regarding migraine triggers associated with diet, fatigue and stress or improving headache-related coping skills and pain management. While more high quality research is still needed to assess the effectiveness of individual manual therapies for the treatment of migraine, understanding the use of these management approaches by chiropractors and their influence on migraine health outcomes, both individually and synergistically, may prove helpful in the design of future clinical trials that aim to assess the overall effectiveness of chiropractic migraine management. Chiropractic clinical trials have yet to incorporate any multimodal aspects of chiropractic care that may influence underlying migraine mechanisms and have been limited to the assessment of unimodal manual therapy interventions for which headache treatment guidelines report only weak evidence or level III recommendations [37, 38].

### Factors associated with high migraine caseload

Our analyses did not identify any practitioner characteristics (practitioner age, gender or place of education) that were associated with a high migraine caseload, suggesting that a broad cross-section of the Australian chiropractors are frequently managing those with migraine. However, our research highlights several practice-setting and clinical management characteristics associated with chiropractors managing a high migraine caseload and which raise valuable questions about the therapeutic or philosophical approaches that may be common to chiropractic migraine management.

Our study found chiropractors with a high migraine caseload were associated with treating spine regions (cervical, thoracic and lumbar) including referred and radicular spine symptoms associated with noxious stimulation of nerve endings and direct nerve root compression respectively [39], as well as treating upper limb disorders. Previous studies report manual therapies, particularly manipulative therapies, to be the most common therapies utilised by chiropractors when treating the spine and upper limb [34, 40–43]. Spinal manipulation in particular is reported to be the most popular treatment modality utlised by Australian chiropractors [35] and the only therapeutic modality to be evaluated by the profession for the treatment of migraine [15]. While unclear from our findings directly, these associations may suggest a greater preference for the use of manual therapies when compared to the use of other therapies amongst chiropractors with a high migraine caseload. More research is needed to assess the use of other therapeutic approaches that may also fall within the scope of chiropractors in their management of migraine. This could include the use of relaxation methods, herbs, minerals, supplements and physical therapies as identified within non-pharmaceutical migraine treatment guidelines [37, 44–46]. More research is also needed to understand the clinical circumstances within which chiropractors decide to refer patients with migraine to other healthcare providers for management and treatment that is outside their scope of practice.

Our analyses identified chiropractors with a high migraine caseload as more likely to provide treatment of patients with non-musculoskeletal conditions. While migraine itself is classified as a neurological disorder, the classification of migraine as a non-musculoskeletal condition is less straight forward when considering evidence of an association with neck pain and the potential role of neck pain in migraine pathophysiology [10, 11, 47, 48]. However, the treatment of a number of non-musculoskeletal conditions with manual therapies by chiropractors is controversial, [49, 50] not least because of the significant methodological limitations in related clinical trials [51, 52] and concerns raised about the lack of biological plausibility to support how manual therapies, such as spinal manipulative therapy (SMT), might influence the underlying pathophysiology of these conditions [53]. On the other hand, higher headache disability and chronicity is more common amongst those who seek complementary medicine including chiropractic [23, 54] and this is associated with greater levels of anxiety and depression [55, 56]. With the interest by some chiropractors toward improving overall patient health, including mental and emotional well-being [35, 57, 58], more research is needed to understand whether the association with treatment of patients with non-musculoskeletal conditions may relate to care that is aimed to assist in the management of common migraine comorbidities, such as anxiety and depression, or toward the management of non-musculoskeletal conditions unrelated to migraine.

Our study also found chiropractors with a high migraine caseload are associated with providing spinal health maintenance and prevention. While there is limited research to identify a universal evidence-based definition of chiropractic maintenance care [59, 60], the role of preventative care is well recognised within healthcare settings including for the prevention of migraine [61], which often presents as a chronic or recurring condition [62, 63]. As such, the need to help sufferers through ongoing support, advice or treatment may be clinically indicated under a prevention paradigm. While ongoing SMT may be a popular component of chiropractic prevention [64, 65], more research is needed to understand all of the therapeutic modalities and approaches utilised under this therapeutic paradigm. With few clinical trials having included sufficient long-term follow-up to assess the benefits of chiropractic spinal health maintenance and prevention, no robust conclusions can be yet made about the long-term outcomes associated with this approach to care both for the management of conditions associated with the spine or the effect this type of care may have on those with migraine.

Our analyses identified chiropractors with a high migraine caseload as more likely to not have a psychologist/counsellor practicing at the same practice location. While psychologists can be a key healthcare provider for those with headache [38, 66, 67] it may be difficult to explain why chiropractors with a high migraine caseload are less likely to practice alongside psychologists. Possible explanations may be the potential influence of existing incentives for greater collaboration and therefore proximity between psychologists and other healthcare providers [68] or the possibility that chiropractors who often manage migraine may have a more independent therapeutic approach to the management of psychological aspects of patient health [69] suggesting less proximity reflects less inter-disciplinary collaboration with psychologists when managing this patient population. Alternatively, this could simply reflect a more general trend for Australian psychologists to work in independent private practice settings [70].

The association with discussing medications (including for pain/inflammation) by chiropractors who often manage migraine raises valuable questions about the nature of these patient discussions. These discussions may reflect the practitioners aim to assist migraine patients to manage their health *‘without the use of drugs or surgery’*, a defining therapeutic and philosophical approach to patient care encouraged by chiropractic political bodies [71, 72] promoting better health without an unnecessary dependence on medications. These discussions may also reflect patient’s raising concerns or dissatisfaction with migraine medications, a finding that has been reported as a key predictor for the use of complementary medicine including chiropractic for this patient population [73, 74]. As a result, discussing current and previous migraine medications may be more common place inside consultations with migraine patients. More research is needed to understand the nature of discussions regarding migraine medications and whether these discussions extend beyond the normal documentation of current and previous treatments for a presenting complaint as expected for registered chiropractors under regulatory guidelines [75].

### Limitations

Our secondary analysis of the ACORN cross-sectional survey provides an opportunity to answer a number of questions and identify further pertinent questions for future enquiry regarding chiropractic migraine management. Drawing strong conclusions from our research may be limited due to our analysis being secondary and the quality and fit of existing data to our research. As such, it cannot be concluded that the associations drawn from this secondary analysis are unique to the management of migraine patients. Our findings rely on practitioners understanding the classification criteria for migraine headache and the retrospective recall of practitioners when answering the original ACORN questionnaire. The Likert categories provided in the ACORN questionnaire (‘never’, ‘rarely’, ‘sometimes’, ‘often’) for the frequency of migraine management are also subject to practitioner interpretation of these terms. There would also be a risk of selection bias if the features of the practitioners responding to the ACORN survey are less than representative of the wider profession. While the associations reported from our secondary analysis of the ACORN cross-sectional survey are preliminary, the findings nevertheless are valuable in helping to generate hypotheses to further explore the management and effectiveness of headache and migraine management by chiropractors.

## Conclusions

Migraine appears to be a significant component of chiropractic caseload. There is a need for more high-quality research to better understand how chiropractors manage this patient population and to understand the prevalence, burden and comorbidities associated with migraine patients who seek help from these providers. Such information is important in helping to inform safe, effective and coordinated care for migraine sufferers within the wider health system.

## Additional file

Additional file 1: ACORN national survey questionnaire (PDF 78 kb)

## Abbreviations

ACORN: Australian Chiropractic Research Network
ARCCIM: Australian Research Centre in Complementary and Integrative Medicine
ATSI: Aboriginal and Torres Strait Islander
HVLA: High Velocity Low Amplitude
MRI: Magnetic Resonance Imaging
PBRN: Practice-Based Research Network
SEMG: Surface Electromyography
SMT: Spinal Manipulative Therapy
YLDs: Years Lived with Disability
