# Supplementary material for: The treatment of migraine patients within chiropractic: analysis of a nationally representative survey of 1869 chiropractors
Source: BMC Complement Altern Med. 2017 Dec 4;17:519. doi: 10.1186/s12906-017-2026-3 (PMC5715542; doi:10.1186/s12906-017-2026-3)
Supplement: Additional file 1: — ACORN national survey questionnaire (PDF 78 kb) [file 12906_2017_2026_MOESM1_ESM.pdf]

ACORN PRACTITIONER QUESTIONNAIRE

Chiropractic practitioner characteristics

Q1 What is your age in years?

☐

Q2 What is your gender?

☐ Male
☐ Female

Q3 Are you currently in private chiropractic practice?

☐ No
☐ Yes, how many years? ☐

Q4 What is the highest level of chiropractic professional qualification that you hold?

☐ Diploma
☐ Masters degree
☐ Advanced Diploma
☐ PhD
☐ Bachelor (or Double Bachelor) degree
☐ Doctor of Chiropractic

Q5 Are you a member of any of the following professional chiropractic organisations? *(select all that apply)*

☐ CAA
☐ CAA and COCA
☐ COCA
☐ None
☐ Other(s) *(Please specify)*

Q6 Indicate all the roles in which you have been involved as a chiropractor over the last 12 months: *(select all that apply)*

☐ University teaching
☐ Research
☐ Clinical supervision
☐ Volunteer work
☐ Private practice
☐ Professional organisation activities

Q7 Do you routinely consult patients in a language other than English?

☐ No
☐ Yes *(Please specify)*

Practice characteristics

Q8 How many of the following would you provide on average, per week?

a) Patient care hours ☐

b) Patient visits ☐

Q9 Do you practice in more than one location?

☐ No
☐ Yes, how many in total ☐

Q10 Indicate all other health professionals working in your practice location(s): *(select all that apply)*

☐ GP
☐ Exercise Physiologist
☐ Podiatrist
☐ Psychologist/Counsellor
☐ Medical specialist
☐ Occupational Therapist
☐ Physiotherapist
☐ None
☐ Another Chiropractor
☐ Other(s) *(Please specify)*

Q11 Do you have a professional referral relationship (sending and/or receiving referrals) with any of the following practitioners: *(select all that apply)*

☐ GP
☐ Medical specialist
☐ Psychologist/Counsellor
☐ Exercise Physiologist
☐ Physiotherapist
☐ None
☐ Occupational Therapist
☐ Other(s) *(Please specify)*
☐ Podiatrist

Q12 In which state/territory do you practice? *(select all that apply)*

NSW	VIC	QLD	WA	SA	TAS	NT	ACT
☐	☐	☐	☐	☐	☐	☐	☐

Q13 Which of the following best describes your practice location(s)? *(select all that apply)*

☐ Urban ☐ Rural ☐ Remote

Q14 How frequently do you use diagnostic imaging as part of your practice?

☐ Never ☐ Rarely ☐ Sometimes ☐ Often

Q15 Indicate all imaging facilities or scanning tools you have on site: *(select all that apply)*

☐ X-ray
☐ Thermography
☐ MRI
☐ None
☐ SEMG
☐ Other(s) *(Please specify)*
☐ Diagnostic Ultrasound

Q16 Indicate when you use electronic records: *(select all that apply)*

☐ Initial History
☐ Examination findings
☐ Subsequent patient visits
☐ Never

Clinical management

Q17 Indicate the frequency with which you discuss the following as part of your care/management plans:

	Never	Rarely	Sometimes	Often
Diet / nutrition	☐	☐	☐	☐
Smoking / Drugs / Alcohol	☐	☐	☐	☐
Physical Activity / Fitness	☐	☐	☐	☐
Occupational Health and Safety	☐	☐	☐	☐
Pain Counselling	☐	☐	☐	☐
Nutritional Supplements (including vitamins, minerals, herbs)	☐	☐	☐	☐
Medication (including for pain / inflammation)	☐	☐	☐	☐
Other *(Please specify)*	☐	☐	☐	☐

Q18 Indicate the frequency with which you treat patients that present with the following conditions:

	Never	Rarely	Sometimes	Often
Neck pain (axial)	☐	☐	☐	☐
Neck pain (referred/radicular)	☐	☐	☐	☐
Thoracic pain (axial)	☐	☐	☐	☐
Thoracic pain (referred/radicular)	☐	☐	☐	☐
Low back pain (axial)	☐	☐	☐	☐
Low back pain (referred/radicular)	☐	☐	☐	☐
Lower limb musculoskeletal disorders (hip, knee, ankle, foot)	☐	☐	☐	☐
Upper limb musculoskeletal disorders (shoulder, elbow, wrist, hand)	☐	☐	☐	☐
Postural disorders (including lordosis, thoracic kyphosis, scoliosis)	☐	☐	☐	☐
Degenerative spine conditions (including spondylolisthesis)	☐	☐	☐	☐
Headache disorders (including cervicogenic, tension)	☐	☐	☐	☐
Migraine disorders	☐	☐	☐	☐
Spinal health maintenance/prevention	☐	☐	☐	☐
Non-musculoskeletal disorders *(Please specify)* ______	☐	☐	☐	☐
Other *(Please specify)* ______	☐	☐	☐	☐

Q19 Indicate the frequency with which you treat the following patient subgroups:

	Never	Rarely	Sometimes	Often
Children (up to 3 years)	☐	☐	☐	☐
Children (4 to 18 years)	☐	☐	☐	☐
Older people (65 years or over)	☐	☐	☐	☐
Aboriginal and Torres Strait Islander people	☐	☐	☐	☐
Pregnant women	☐	☐	☐	☐
Athletes or sports people	☐	☐	☐	☐
People with work-related injuries	☐	☐	☐	☐
People with traffic-related injuries	☐	☐	☐	☐
People receiving post-surgical rehabilitation	☐	☐	☐	☐
Non-English speaking ethnic group(s) *(Please specify)* ______	☐	☐	☐	☐
Other *(Please specify)* ______	☐	☐	☐	☐

Q20 Indicate the frequency with which you employ the following Techniques/Methods in your patient management:

	Never	Rarely	Sometimes	Often
Drop-piece techniques / Thompson® or similar	☐	☐	☐	☐
Biomechanical pelvic blocking / Sacro-Occipital Technique®	☐	☐	☐	☐
Instrument adjusting	☐	☐	☐	☐
Chiropractic BioPhysics®	☐	☐	☐	☐
High velocity, low amplitude adjustment / manipulation / mobilisation	☐	☐	☐	☐
Applied Kinesiology® (AK)	☐	☐	☐	☐
Flexion-distraction	☐	☐	☐	☐
Functional Neurology	☐	☐	☐	☐
Extremity manipulation	☐	☐	☐	☐
Other technique or intervention *(Please specify)* ______	☐	☐	☐	☐

Q21 Indicate the frequency with which you employ the following Musculoskeletal Interventions in your patient management:

	Never	Rarely	Sometimes	Often
Dry needling or Acupuncture	☐	☐	☐	☐
Soft tissue therapy, trigger point therapy, massage therapy, stretching	☐	☐	☐	☐
Electro-modalities (TENS, laser, interferential/ultrasound therapy)	☐	☐	☐	☐
Heat / cryotherapy	☐	☐	☐	☐
Orthotics (foot care)	☐	☐	☐	☐
Specific exercise therapy / rehabilitation / injury taping	☐	☐	☐	☐
Other *(Please specify)* ______	☐	☐	☐	☐
